# Pilot Studies on Empathy and Closeness in Mutual Entrainment/Improvisation vs. Formalised Dance with Different Types of Rhythm (Regular, Irregular, and No Rhythm) and Coupling (Visual, Haptic, Full Coupling): Building a Case for the Origin of Dance in Mutual Entrainment Empathic Interactions in the Mother–Infant Dyad

**DOI:** 10.3390/bs13100859

**Published:** 2023-10-20

**Authors:** Tudor Balinisteanu

**Affiliations:** 1 Neuroaesthetics Lab, University of Suceava, 720229 Suceava, Romania; tudor@texmar.ro; 2 Psychology Department, Goldsmiths, University of London, London SE14 6NW, UK

**Keywords:** origins of dance, empathy, closeness, entrainment, mutual entrainment, partnered dance, mother–infant dyad

## Abstract

This paper employs a novel research design to examine changes in empathy and closeness in partnered face-to-face dance, considering both different types of rhythm (regular, irregular, and no external rhythm, or ‘mutual entrainment only’) and different types of coupling (visual only, haptic only, and full visual and haptic coupling). Two studies were undertaken to pilot the design. In both studies, the Interpersonal Reactivity Index and Inclusion of Other in the Self were used to measure empathy and closeness, respectively. Study 1 employed 24 participants (12 pairs) distributed across two rhythm conditions, external regular rhythm, and no external rhythm, with full coupling in both conditions. Closeness increased similarly in both conditions. Empathic concern (EC) was significantly affected in the ‘no rhythm’ condition. Study 2 employed 54 participants assigned to form pairs and distributed across all combinations of rhythm and coupling types. Closeness decreased with irregular rhythm. EC increased in the ‘no rhythm’ conditions relative to regular rhythm. Fantasy (F) decreased with haptic coupling only (no visual coupling) while personal distress (PD) increased. In addition, the analyses suggest that perspective taking (PT) increases with irregular rhythm and in the condition without rhythm (mutual entrainment only). The discussion gauges the value of the designs and results for capturing changes in empathy and closeness with different rhythm and coupling types. Capturing such changes is important for research on the origins of dance in empathic mutual entrainment in the mother–infant dyad.

## 1. Introduction

Studies of the different ways in which various rhythm types combined with various coupling types affect closeness and empathy in partnered dance could shed light on the origins of dance in both evolutionary and developmental perspectives. For example, one may hypothesise that mutual entrainment in the absence of external rhythm precedes entrainment to regular and irregular rhythm on both evolutionary and developmental scales. Likewise, it may be hypothesised that full coupling in movement together precedes ‘visual coupling only’ on same scales. Affective empathy and closeness are ubiquitous in early infancy, where the predominant form of moving together involves mother and infant. It is more likely that ‘visual coupling only’ is predominant in face-to-face partnered movement in adult life, suggesting that cognitive empathy is predominant in such interactions. A research design that could capture the ways in which empathy and closeness depend on combinations of types of coupling and types of rhythm (or its absence), could shed light on the developmental and evolutionary origins of dance, helping to test the theory that dance originates from movement together in the mother–infant dyad; it could further test whether dance evolved as a means to formalise partnered movement in order to preserve the prosocial benefits of empathy and closeness experienced in infancy (developmentally speaking) and among ancestral humans prior to the full development of executive control capacity (evolutionarily speaking).

This theory could be tested by formulating the following set of hypotheses, by no means exhaustive: full coupling with mutual entrainment (no external beat) should increase affective empathy more than full coupling with an external regular rhythm. Conversely, full coupling with an external regular rhythm should increase cognitive empathy more than full coupling with mutual entrainment without an external rhythm. If cognitive empathy and affective empathy are seen as complementary dimensions of empathy, affective empathy should decrease with regular rhythm, while simultaneously, cognitive empathy should increase. While such results would be consistent with a developmental hypothesis that posits the origins of dance in mother–infant mutual entrainment that later becomes formalised movement together, face-to-face, in pairs, with external rhythm [1,2], they might also support an evolutionary hypothesis that reflects the developmental hypothesis. To elaborate, ancestral humans may have first discovered the benefits of mutual entrainment in terms of increasing affective empathy [3,4], an experience that, later, may have been societally formalised in order to preserve the benefits of empathy for prosocial behaviour within evolving small-scale societies, e.g., [5,6,7,8,9,10]. As the brain’s capacity for executive control evolved and developed, affective empathy engendered by mutual entrainment may have yielded some of its power in favour of cognitive empathy. In this view, the evolutionary hypothesis posits that dance evolved from mutual entrainment as opposed to entrainment to external beats.

## 2. Materials and Methods

### 2.1. Design

All experimental work was approved by the Ethics Committee of Goldsmiths, University of London.

The research design proposed here is based on research designs employed in two studies. In a study of group dance synchronisation by Chauvigné et al. [11], the participants danced in a circle to an external beat with haptic and visual coupling. The dancers primarily used haptic coupling to synchronise. In an fMRI study by Chauvigné, Belyk and Brown [12], participants inside the scanner used haptic coupling to follow or lead the movements of a researcher sitting next to the magnet (a condition with haptic coupling only, and no external rhythm pattern). In a comparison condition, both participant and researcher followed a pre-learned pattern; leading and following was thus minimised. Mentalising and social reward were stronger in the latter condition. The research design proposed here takes forward the idea of disentangling types of coupling as well as the opposition between formalised pattern and improvisation (or mutual entrainment) to propose a focus on dynamics of empathy; empathy is likely present in synchronised face-to-face partnered movement when participants also use mentalising, an experience that is overall socially rewarding.

The first version of the research design (Design 1) presented here employed two conditions, as indicated in Table 1. This design allows for a comparison of the effect of formalised movement on a regular beat with the effect of mutual entrainment in the absence of external rhythm. The types of coupling are listed as separate columns in order to emphasise that mutual entrainment as well as entrainment to external rhythm can both take place, either with full coupling or with only one type of coupling. The participants were placed in pairs face-to-face, and asked to either follow an external regular beat or improvise without any external sound, with full coupling (looking at each other and holding hands). The design was tested as a between-subjects design to avoid familiarisation with the task and a build-up of empathy between partners.

Experiment 1 tested Design 1 between subjects with the two rhythm types: regular rhythm and rhythm resulting from mutual entrainment only, using the Inclusion of Other in the Self (IOS) [13] and the Interpersonal Reactivity Index (IRI) [14] instruments to measure closeness and empathy. The participants were asked to move in a simple side-to-side step, face-to-face, with eyes open and holding hands, for 7 min. The IOS and IRI were administered before and after the intervention. During the time in which they filled in the questionnaires, the participants were seated face-to-face in the same pairs they formed for moving together. There were 6 pairs in each condition (24 participants).

The data were analysed by inputting the result of subtractions of the pre-test scores from post-test scores into the one-way ANOVA model separately for the IOS scores and each of the IRI subscales. A within-between ANOVA model was used to analyse pre- and post-test scores between subjects, with time as a within-subjects factor.

The research design described above was further elaborated to disentangle the effects of the type of coupling (full, visual only, and haptic only) from the type of rhythm (external regular rhythm, external irregular rhythm, and mutual entrainment only) (see Table 2 below). Hence, this research design (Design 2) uses 2 IVs, each with 3 levels: rhythm (regular, irregular, improvisation) and coupling (full, visual only, and haptic only), and the same 2 DVs (IRI measured empathy and IOS measured closeness).

Experiment 2 tested Design 2 between subjects with all possible combinations of rhythm and coupling. The 9 conditions can be divided into 3 sets of 3:

Set 1: a simple side-to-side step movement on a 4/4 rhythm with:

(1a) Full interpersonal feedback (+haptic, +vision, +regular rhythm);

(1b) Visual coupling only (−haptic, +vision, +regular rhythm);

(1c) Haptic coupling only (+haptic, −vision, +regular rhythm);

Set 2: a simple side-to-side step movement on a 7/8 rhythm with:

(2a) Full interpersonal feedback (+haptic, +vision, +irregular rhythm);

(2b) Visual coupling only (−haptic, +vision, +irregular rhythm);

(2c) Haptic coupling only (+haptic, −vision, +irregular rhythm);

Set 3: a simple side-to-side step movement without external formal rhythm (improvisation or ‘mutual entrainment only’):

(3a) Full interpersonal feedback (+haptic, +vision, −auditory coupling);

(3b) Visual coupling only (−haptic, +vision, −auditory);

(3c) Haptic coupling only (+haptic, −vision, −auditory).

The DVs, closeness and empathy, were again measured using the IOS scale and the 4 IRI subscales, respectively. As before, the participants were randomly allocated in evenly split pairs (3 pairs per condition, 54 participants in total), and the interaction lasted 7 min. IOS and IRI scores were collected before and after the trials. The same soundtracks were used. While completing the questionnaires, the participants were seated face-to-face at opposite sides of a table, in the same pairs that they were asked to form for the interaction.

A factorial ANOVA was performed on the difference between IOS post-test scores and IOS pre-test scores and also on the difference between IRI post- and pre-test scores for each of the four subscales separately. In addition, mixed repeated measures with within-between ANOVAs were performed using the pre-test and post-test scores, with time as a within-subjects factor. Linear regressions were used to determine whether pre-test IOS scores predict variation (measured as the value that results from subtracting pre- from post-test scores) on the IRI subscales. Similarly, linear regressions were used to determine whether pre-test IRI subscales values predict variation in closeness.

### 2.2. Participants

#### 2.2.1. Experiment 1 (Design 1)

For Experiment 1, the GPower 3.1 freeware was used to perform an a priori power analysis in order to estimate the required participants sample size for two-tailed hypotheses and equal numbers in each group. For the one-way ANOVA, the desired effect size f was set at 0.333 based on a partial eta squared (ƞ^2^) value of 0.1. The probability of alpha error was set at 0.05, the desired power at 0.80, and the number of groups was 2. GPower estimated that a sample size of 74 participants would be required for these parameters. For a within-between repeated measures analysis with ƞ^2^ = 0.1, alpha = 0.05, power = 0.8, 2 groups, 2 measurements, a conservative value of 0 for the correlation among repeated measures, and a non-sphericity correction value of 1 (hence assuming that the sphericity assumption would be met), GPower indicated a required effect size f of 0.333 and a total sample size of 38 participants.

However, it was only possible to recruit 24 participants of mixed nationalities (3 males, 21 females, aged 22–39, M = 26 years, SD = 4.222). The participants were recruited using pages of Facebook groups based in London, UK, as well as posters, email lists, and Facebook groups internal to Goldsmiths College, University of London. All participants received a small remuneration for their participation.

#### 2.2.2. Experiment 2 (Design 2)

For Experiment 2, the GPower calculation of the estimated required sample size was 113 participants for a factorial ANOVA with ƞ^2^ set at 0.1, yielding an effect size f = 0.333, and with alpha = 0.05, power = 0.80, numerator df = (3 − 1) × (3 − 1) = 4, and 9 groups. For a within-between repeated measures analysis with ƞ^2^ set at 0.1, yielding an effect size f = 0.333, and with alpha = 0.05, power = 0.80, 9 groups, number of measurements [(9 − 1) × (2 − 1)] + 1 = 9, correlation between repeated measures at 0, and a non-sphericity correction value of 1, GPower indicated an estimated required sample size of 81.

However, it was only possible to recruit 54 participants (3 males, 51 females, aged 18–50, M = 21.78 years, SD = 5.276). The participants were recruited from among advanced English Language and Literature students at University of Suceava and received course credits for their participation. The participants were predominantly Romanian, but the sample included other nationalities as well.

### 2.3. Materials

The Interpersonal Reactivity Index (IRI) is a powerful tool for disentangling the effects of affective empathy and cognitive empathy because two of its subscales measure affective empathy (the empathic concern and personal distress subscales), while the other two measure cognitive empathy (the perspective taking and fantasy subscales). The IRI items could be modified to work as a measure of state empathy instead of trait empathy, but in testing the designs proposed here, the original IRI has been used. The participants were asked to focus on their feelings ‘in the present moment’ when scoring the IRI items, and The Inclusion of Other in the Self (IOS) instrument could be used to verify, for example, whether affective empathy engenders stronger closeness than cognitive empathy. Here, the IOS was used to merely gauge the effect of various rhythm and coupling type combinations on closeness.

The Inclusion of Other in the Self (IOS) test is a single-item pictorial, seven-step interval-level scale representing 7 ways in which two selves may overlap, arranged progressively from no overlap to, by degrees, significantly overlapping. According to Aron, Aron, and Smollan [13], it is intended to measure the feeling of closeness, theorised as a common theme of interpersonal connectedness emerging from various research on empathy, intimacy, and inter-subjectivity. Since it measures a feeling, the IOS is a state measure. In this study the circles were assigned values from 0 to 6, with 0 for no overlap and 6 for the strongest overlap. When filling in the test, in order to facilitate the participants’ assessment of self–other overlap, they were seated across from each other at a table in the same pairs that were later formed for the dance interaction.

The Interpersonal Reactivity Index scale is a 28-item questionnaire comprising 4 subscales, each with 7 items [14]. The subscales measure 4 different dimensions of dispositional empathy: empathic concern measures the feeling of compassion for another individual in distress; perspective taking measures cognitive, as opposed to affective, empathy; personal distress measures one’s self-focused ability to empathise with another individual in distress; and fantasy measures empathy for fictional characters, such as may be encountered in the narrative of a book or film. The response to each item is recorded on a Likert scale with two anchors (A = does not describe me well; E = describes me very well). In the present study, A was assigned a value of 0 and E was assigned a value of 4. The IRI instrument measures general empathy on each of these subscales and is not intended as a means of assessing total empathy (i.e., one should not analyse the sum of the scores obtained on each of the 4 subscales, but the scores on each subscale separately). Internal consistency (alpha) coefficients range from 0.68 to 0.79 [15].

The soundtracks, one with a 4/4, the other with a 7/8 rhythm, were obtained using the GarageBand 2.3.6 software (Supplementary Materials S1 and S2). Only a drum sound was programmed on the soundtracks. No other instrument was present. The drum sound was an electronic rendering of the sound made by a conga drum. This instrument selection was guided by the assumption that drums were among the very first instruments available to humans, being present in our daily lives from ancient times. The soundtracks were 7 min long.

### 2.4. Procedure

At the beginning of each trial, the experimenter offered an outline of the session activities. Each participant was then randomly allocated a seat facing another participant seated directly across a table. The two participants thus seated formed a pair, the same pair which they subsequently formed for the dance interaction. The participants were then instructed to fill in their responses to the IRI and IOS questionnaires, with IRI responses requested first. Informed consent was obtained at this stage.

After this pre-trial phase, the participants were invited onto the dance floor as partners in their pairs. In all cases, this was a classroom floor, roughly similar in both experiments in terms of muffling the sound made by the participants’ feet, an important consideration for the conditions with improvisation (mutual entrainment only, without external sound). The participants were instructed to do a simple side-to-side step movement, as if they were dancing at a country fair or a casual party. Each interaction was timed and lasted 7 min. In the interactions with external sound, the sound was played using an audio system with high-power speakers in the first experiment, and an audio system with medium-power speakers in the second experiment. However, the sound power was similar in both experiments because the difference in power in the second experiment was supplemented by the use of a subwoofer. The same audio player was used in both experiments.

In the post-trial phase, the participants were requested to complete another set of IRI and IOS questionnaires. At the end of the session, the participants were informed that they could request a briefing about the study results obtained after all the experiment sessions had been completed and the data were analysed. After calculating the scores at pre- and post-trial points, the data were inputted into an IBM SPSS database and analysed using SPSS software. The data are also available in Excel as Supplementary Materials S3 and S4A–E.

## 3. Results

### 3.1. Experiment 1 (Design 1)

The experiment was run with 24 participants (3 males, 21 females, aged 22–39, M = 26 years, SD = 4.222). The IV was **rhythm** (2 levels: regular and improvisation without sound, or ‘mutual entrainment only’), and the DVs were measured using the IRI and IOS scales.

#### 3.1.1. Analyses of Closeness (IOS Scores)

The mean (M) of the improvisation (mutual entrainment only) scores obtained by subtracting the pre-trial scores from the post-trial scores was 1.083, standard error (SE) = 0.228, 95% confidence intervals (CI). For formal regular rhythm, M = 1, SE = 0.443, CI = 95%. A factorial ANOVA, between-subjects, DV = IOSpost − IOSpre scores, yielded no significant main effect of rhythm, although the mean for closeness with improvisation (mutual entrainment only) was slightly higher than the mean for closeness with formal external regular rhythm.

**Figure 1:**
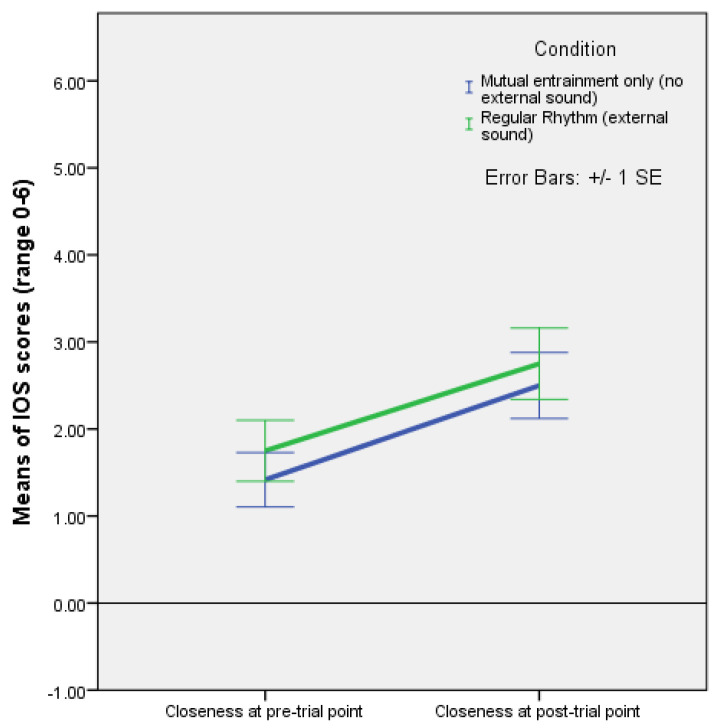
Plot of means with error bars (+/−1 SE) for IOS (closeness) scores (range 0–6) at pre- and post-test points with improvisation (no external sound, mutual entrainment only) and formal regular rhythm (external sound). Dancing to a regular rhythm as well as improvising (mutual entrainment only, without external sound) while holding hands and seeing each other increased closeness, with a significant within-subjects effect of time F(1,22) = 17.405, *p* < 0.001, and partial ƞ^2^ = 0.442 (Experiment 1).

A mixed ANOVA with time as a within-subjects factor, and rhythm as a between-subjects factor found a significant effect of time on closeness F(1,22) = 17.405, *p* < 0.001, and partial ƞ^2^ = 0.442 (Figure 1).

#### 3.1.2. Analyses of Empathy (IRI Scores)

##### IRI-Empathic Concern (EC) Scale

For improvisation (mutual entrainment only), M = 0.1667, SE = 0.824, CI = 95%. For formal regular rhythm, M = −0.75, SE = 0.494, CI = 95%.

A Factorial ANOVA, between-subjects, DV = IRIpost − IRIpre, found no significant main effect of rhythm, although the EC mean with improvisation (mutual entrainment only) was higher than the EC mean with formal regular rhythm.

**Figure 2:**
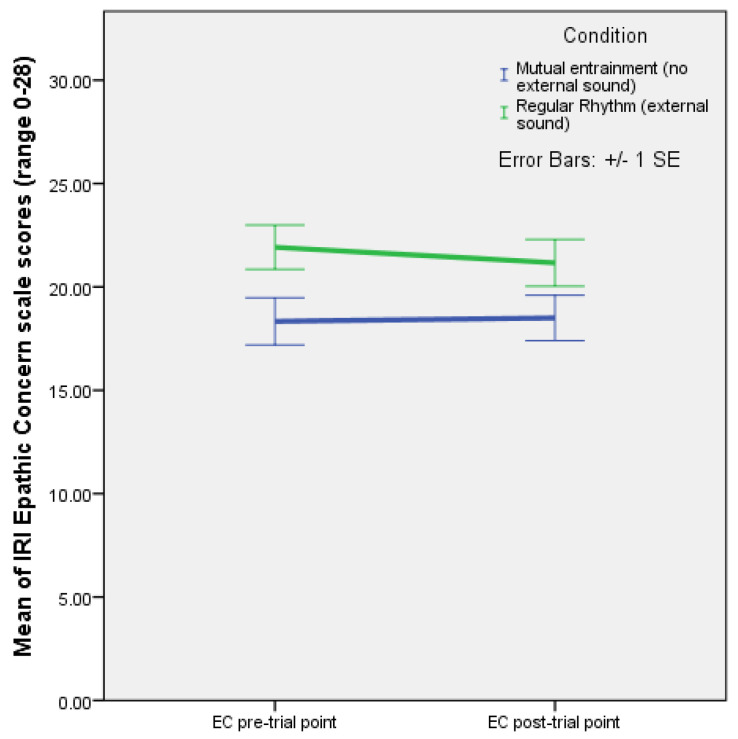
Plot of means with error bars (+/−1 SE) for IRI empathic concern scale (range 0–28) pre- and post-test values with improvisation (mutual entrainment only, no external sound) and regular rhythm (external sound) (Experiment 1).

A mixed ANOVA with time as a within-subjects factor showed a significant effect of rhythm on empathic concern F(1,22) = 4.375, *p* = 0.048, and partial eta squared (ƞ^2^) = 0.166. The plot of estimated marginal means (EMMs) showed that empathic concern increased in the condition with improvisation (mutual entrainment only) and decreased with regular rhythm (see Figure 2).

##### IRI-Fantasy (FS) Scale

For improvisation (mutual entrainment only) scores, M = −0.416, SE = 0.583, and CI = 95%. For formal regular rhythm, M = 0.25, SE = 1.268, and CI = 95%. The factorial ANOVA showed no significant main effect of rhythm, although the FS mean with improvisation (mutual entrainment only) was lower than the FS mean with formal regular rhythm. A mixed ANOVA with time as a within-subjects factor yielded no significant results.

##### IRI-Personal Distress (PD) Scale

For improvisation (mutual entrainment only), M = −0.916, SE = 0.811, and CI = 95%. For formal regular rhythm, M = −1.333, SE = 1.123, and CI = 95%. The factorial ANOVA yielded no significant main effect of rhythm, although the PD mean with improvisation (mutual entrainment only) was higher than the PD mean with formal regular rhythm. A mixed ANOVA with time as a within-subjects factor yielded no significant results.

##### IRI-Perspective Taking (PT) Scale

For improvisation (mutual entrainment only), M = 0.333, SE = 0.71, and CI = 95%. For formal regular rhythm, M = 1.333, SE = 0.541, and CI = 95%. There was no significant main effect of rhythm using the factorial ANOVA, and a mixed ANOVA with time as a within-subjects factor yielded no significant results.

#### 3.1.3. Regressions

Only significant results are reported. Empathic concern pre-test values significantly predicted a variation in closeness with regular rhythm (β = 0.766, *p* = 0.004): model: IOS = −5.971 + 0.318 ECPre (tolerance = 1, VIF = 1, condition index 1–12.441), R^2^ = 0.587, F(1,10) = 14.224, *p* = 0.004. The higher the EC values at the pre-test point, the stronger the increase in closeness when regular rhythm was present. Closeness pre-test values significantly predicted variation in EC with regular rhythm (β = −0.579, *p* = 0.049): model: EC = 0.677 − 0.815 IOSPre (tolerance = 1, VIF = 1, condition index 1–3.31), R^2^ = 0.335, F(1,10) = 5.034, *p* = 0.049). The higher the closeness values at the pre-test point, the weaker the increase in EC when regular rhythm was present.

### 3.2. Experiment 2 (Design 2)

Data were collected from 54 participants (3 males, 51 females, aged 18–50, M = 21.78 years, SD = 5.276). There were 2 IVs: **rhythm**, with 3 levels: regular, irregular, and no external rhythm (i.e., improvisation with mutual entrainment, or internal rhythm only); and **coupling**, with 3 levels: visual and no haptic, visual and haptic, no visual and haptic. The DVs were measured on the IRI and IOS scales.

#### 3.2.1. Analyses of Closeness (IOS Scores)

**Figure 3:**
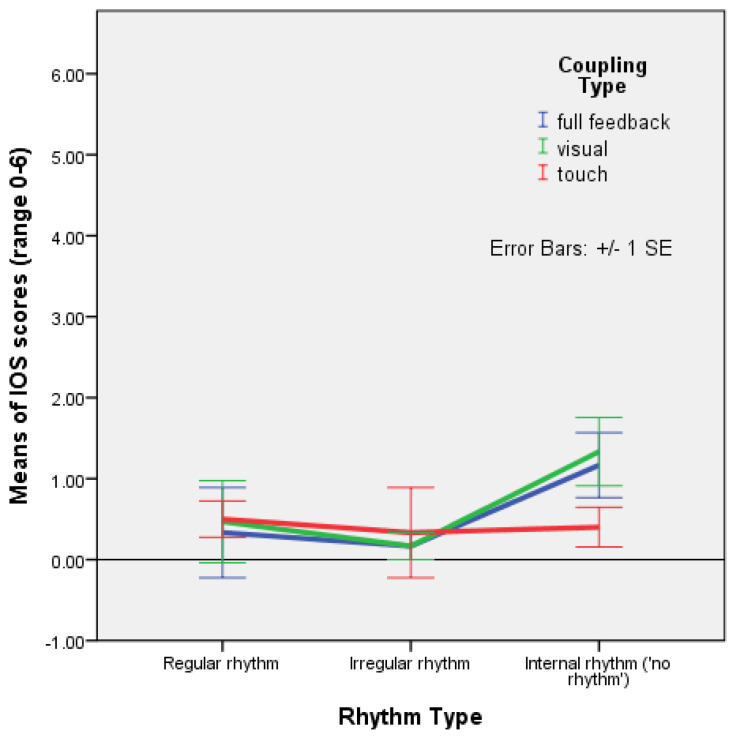
Plot of means for the difference between IOS post- and pre-test values with error bars (+/−1 SE) in the conditions with regular rhythm, irregular rhythm, and improvisation (internal rhythm from mutual entrainment only) with full feedback, and visual and haptic coupling (Experiment 2).

Using a factorial ANOVA, between-subjects, DV = IOSpost − IOSpre scores yielded no significant main effect of rhythm, coupling, and the interaction of rhythm with coupling, on closeness, but the main effect of rhythm looked promising with an encouraging *p* value and a large effect size: F(2,43) = 2.736, *p* = 0.076, and partial ƞ^2^ = 0.111. The profile plot indicates that closeness was stronger when participants held hands with their eyes closed (the condition with haptic coupling only), than when they held or did not hold hands with their eyes open on regular and irregular rhythms. Closeness was weaker in improvisation movement (mutual entrainment only) with haptic coupling only (Figure 3).

There was a significant simple effect of visual coupling (*p* = 0.042) and a near-significant simple effect of full feedback (*p* = 0.079) on the positive mean difference between improvisation (mutual entrainment only) and formal irregular rhythm, showing that closeness was significantly stronger with improvisation than with formal irregular rhythm in the conditions with visual coupling and full feedback. Although not statistically significant, on average, closeness was stronger with improvisation than with formal rhythm, and when rhythm was present, closeness was stronger with regular rhythm than with irregular rhythm (Table S1 in Supplementary Material S5).

**Figure 4:**
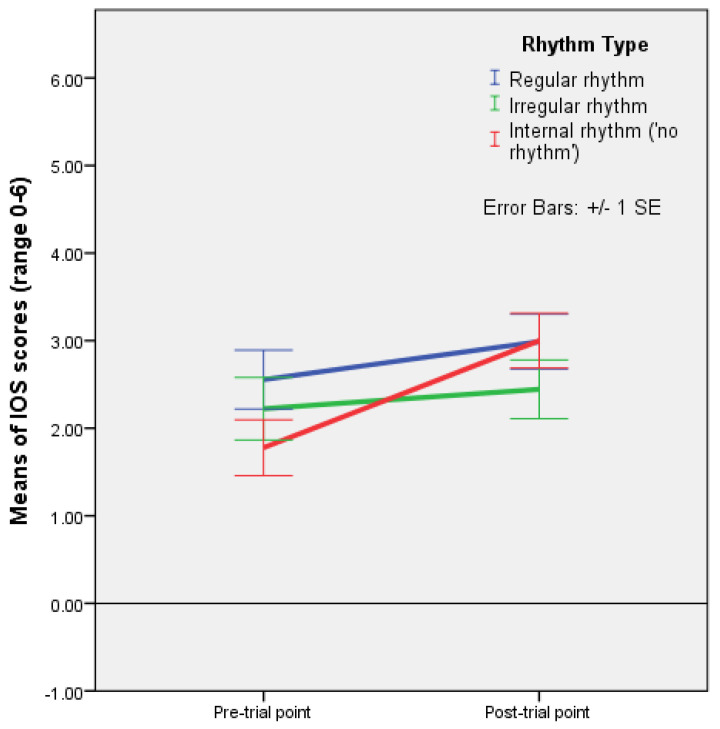
Plot of means for IOS scale values with error bars (+/−1 SE) for regular rhythm, irregular rhythm, and improvisation (internal rhythm) at pre-trial and post-trial points (Experiment 2).

**Figure 5:**
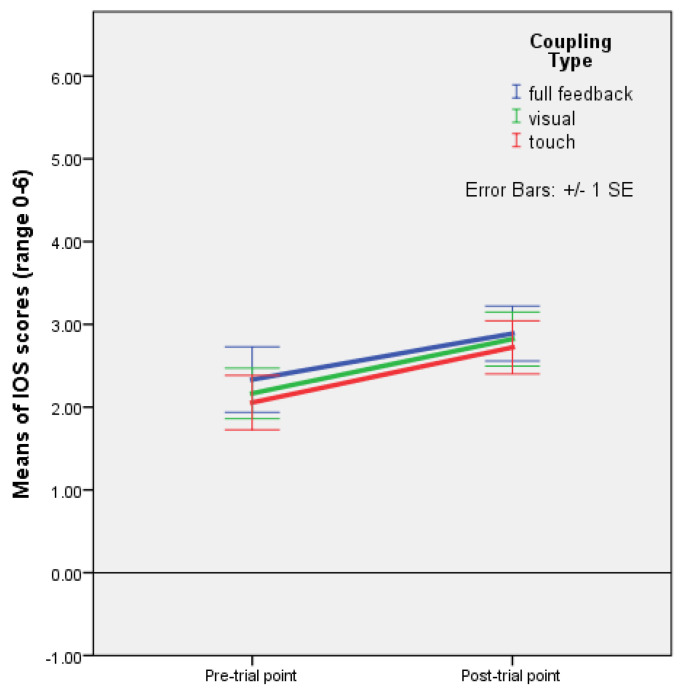
Plot of means for IOS scale values with error bars (+/−1 SE) for full feedback, and visual and haptic coupling at pre-trial and post-trial points (Experiment 2).

A mixed ANOVA with time as a within-subjects factor, and rhythm and coupling as between-subjects factors, showed a significant effect of the interaction between rhythm and time, F(2,45) = 3.842, *p* = 0.029, and partial ƞ^2^ = 0.146. The profile plot shows that closeness increased from pre-interaction to post-interaction points in all conditions, with the highest increase in the condition with improvisation (mutual entrainment only) (see Figure 4, Table 3, and Table S2 in Supplementary Material S5). When rhythm was present, the increase was higher with regular rhythm than with irregular rhythm. As regards the coupling conditions, the profile plot suggests that there is no major difference in increases in closeness by coupling type (Figure 5).

#### 3.2.2. Analyses of Empathy (IRI Scores)

##### IRI-empathic concern (EC) Scale

**Figure 6:**
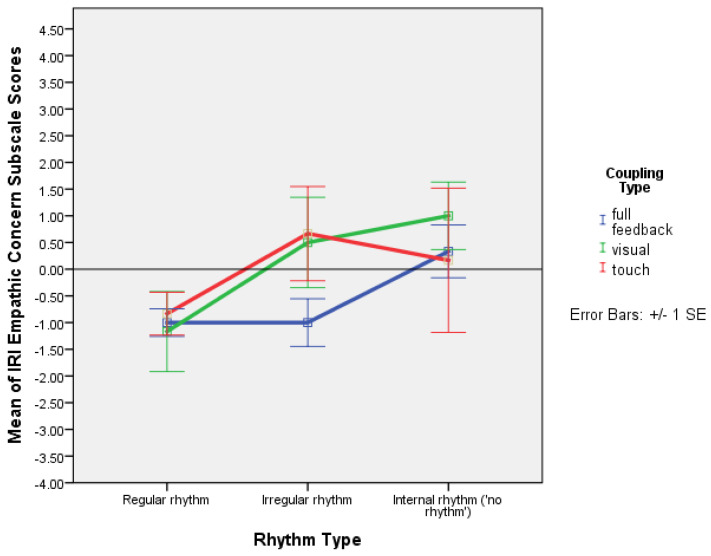
Plot of means with errors bars (+/−1 SE) for the difference between post and pre-test IRI empathic concern scale values for the conditions with regular rhythm, irregular rhythm, and improvisation (‘internal rhythm’ or mutual entrainment) with full feedback, and visual and haptic coupling (Experiment 2).

The factorial ANOVA, between-subjects, DV = IRIpost-IRIpre scores, showed a near-significant main effect of rhythm on empathic concern: F(2,43) = 3.140, *p* = 0.053, and partial ƞ^2^ = 0.127. The post hoc mean difference comparisons showed a near-significant difference (*p* = 0.055) between the condition with regular rhythm and mutual entrainment, and the condition with improvisation (mutual entrainment only), with the higher mean for improvisation (Table 4). The rhythm by coupling comparison of means and the plot show that this difference occurred with visual coupling (Table 5 and Figure 6).

A mixed ANOVA with time as a within-subjects factor yielded no significant result.

##### IRI-Fantasy (FS) Scale

A factorial ANOVA, between-subjects, DV = IRIpost − IRIpre scores showed no significant main effect.

**Figure 7:**
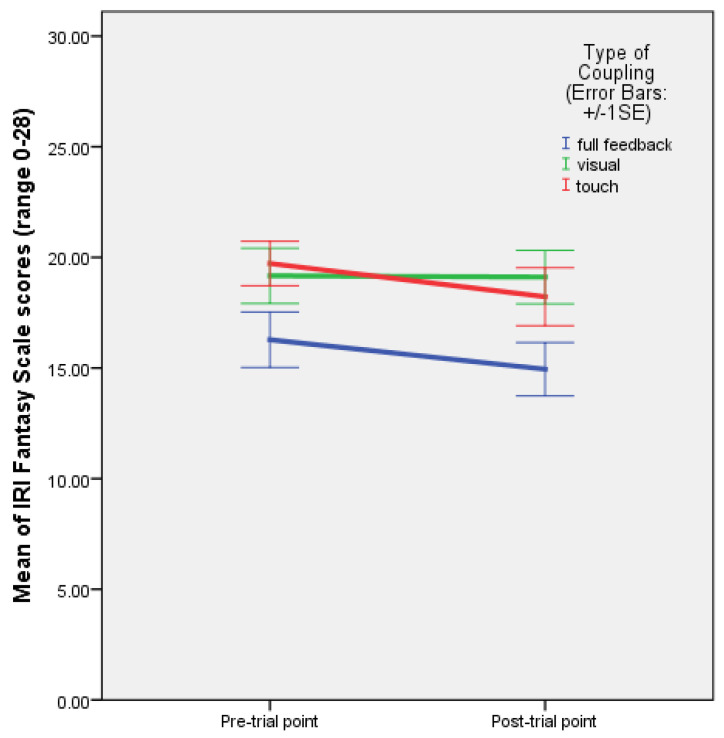
Plot of means with error bars (+/−1 SE) for IRI fantasy scale (range 0–28) pre- and post-test values with full feedback, and visual and haptic coupling (Experiment 2).

Using a mixed ANOVA with time as a within-subjects factor, and rhythm and coupling as between-subjects factors, showed a significant effect of coupling on fantasy: F(2,45) = 3.212, *p* = 0.050, and partial ƞ^2^ = 0.125. The EMMs plot showed that fantasy decreased with ‘haptic coupling only’ and full feedback while staying roughly the same with visual coupling (Figure 7).

##### IRI-Personal Distress (PD) Scale

**Figure 8:**
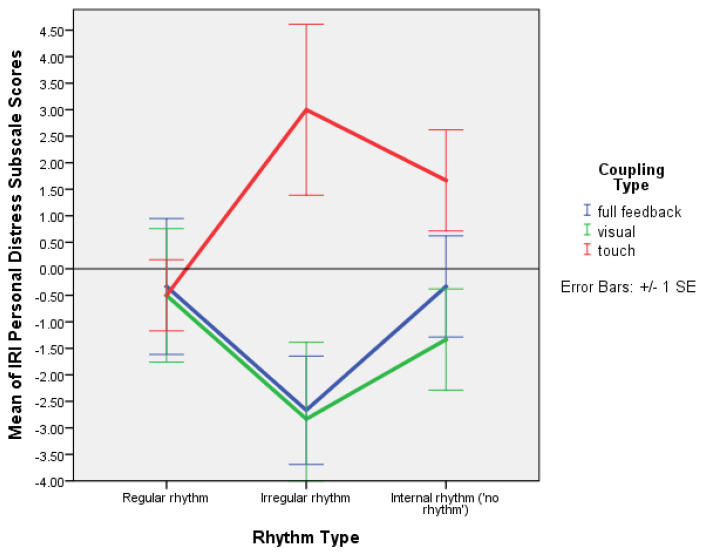
Plot of means for IRI personal distress scale values with error bars (+/−1 SE) for regular rhythm, irregular rhythm, and improvisation (internal rhythm, or no external rhythm) with full feedback, and visual and haptic coupling (Experiment 2).

The factorial ANOVA, between-subjects, DV = IRIpost − IRIpre scores, showed a significant main effect of coupling on personal distress: F(2,45) = 5.599, *p* = 0.007, and partial ƞ^2^ = 0.199. Post hoc mean difference comparisons showed a significant difference between the condition with haptic coupling and both the condition with visual coupling and that with full coupling (Table 6). The most significant difference was between haptic and visual coupling (*p* = 0.009). The multiple comparisons of means and the profile plot show that PD was strongest with irregular rhythm and haptic coupling, and quite strong with improvisation and haptic coupling (Table 7 and Figure 8). The highest PD mean was for the condition in which partners held hands with their eyes closed with an irregular rhythm, less with improvisation, and lesser with regular rhythm.

The simple effects table confirmed that when partners held hands with their eyes closed, the difference between feeling distressed because of one’s partner situation with a regular compared to an irregular rhythm was significant (*p* = 0.039) and higher for irregular rhythm (Table 8).

**Figure 9:**
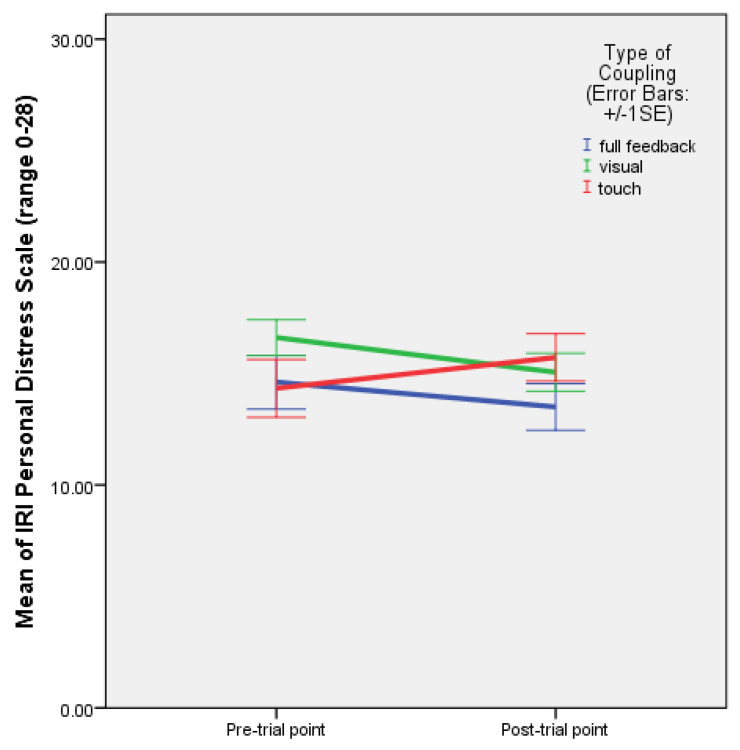
Plot of means with error bars (+/−1 SE) for IRI personal distress scale pre- and post-test values with full feedback, and visual and haptic coupling (Experiment 2).

The mixed ANOVA with time as a within-subjects factor, and rhythm and coupling as between-subjects factors, showed a significant effect of the interaction between time and coupling on personal distress: F(2,45) = 5.599, *p* = 0.007, and partial ƞ^2^ = 0.199. The EMMs plot showed that personal distress increased when partners held hands with their eyes closed, and decreased in the other two coupling conditions (Figure 9).

##### Analysis for IRI-Perspective Taking (PT) Scale

**Figure 10:**
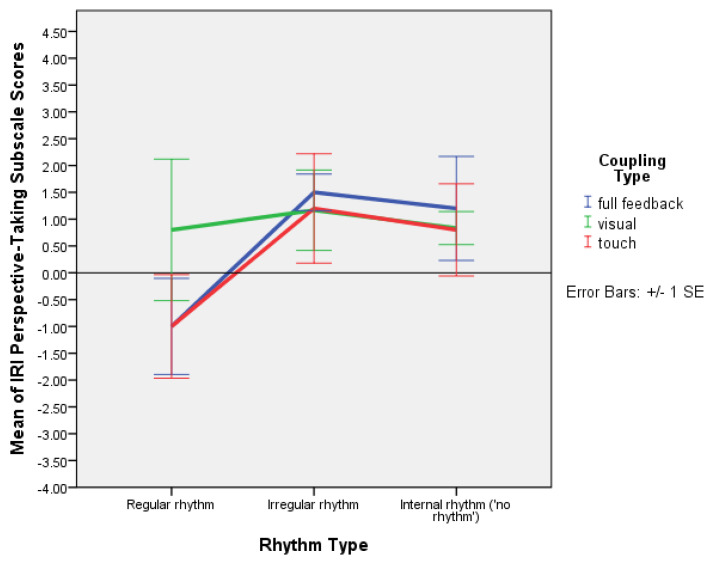
Plot of means for IRI perspective taking scale values with error bars (+/−1 SE) for regular rhythm, irregular rhythm, and improvisation (internal rhythm from mutual entrainment, without external rhythm) with full feedback, and visual and haptic coupling (Experiment 2).

The factorial ANOVA, between-subjects, DV = IRIpost − IRIpre scores, showed a significant main effect of rhythm on perspective taking, F(2,40) = 3.293, *p* = 0.047, and partial ƞ^2^ = 0.141. There was a significant difference in PT between regular and irregular rhythm (*p* = 0.042), as shown in the post hoc multiple comparisons table (Table 9). The multiple comparisons table and the EMMs plot taken together show that perspective taking scores were much higher for irregular rhythm than for regular rhythm, and also higher for improvisation (mutual entrainment) than for regular rhythm, except in the condition with visual coupling only (see Figure 10). A mixed ANOVA with time as a within-subjects factor yielded no significant result.

#### 3.2.3. Regressions

Only significant results are reported. Closeness pre-test values significantly predicted variation in EC with regular rhythm and visual coupling only (β = 0.830, *p* = 0.041): model: EC = 1.0385 + 0.371 IOSPre (tolerance = 1, VIF = 1, condition index 1–4.211), R^2^ = 0.689, F(1,4) = 8.879, *p* = 0.049. The higher the IOS values at the pre-test point, the stronger the increase in EC when regular rhythm was present and partners could see each other without touching. EC pre-test values significantly predicted variation in closeness with mutual entrainment and haptic coupling only (β = −0.853, *p* = 0.031): model: IOS = 7.147–0.309 ECPre (tolerance = 1, VIF = 1, condition index 1–8.036), R^2^ = 0.728, F(1,4) = 10.771, *p* = 0.031. The higher the EC values at the pre-test point, the weaker the increase in closeness with mutual entrainment while participants held hands with their eyes closed.

## 4. Discussion

Most infants begin to produce dance behaviour at about 12 months old, and by their second year of life, they begin to show degrees of cognitive control (e.g., by incorporating learned movement patterns in their dance) [16]. Almost all parents dance with their children during this time. The mother–infant ‘dyadic dance’ involves mutuality, reciprocity, attunement, contingency, coordination, matching, mirroring, reparation, and synchrony [17]. These processes require complex affective and cognitive involvement and are adequately captured by the construct of empathy (affective and cognitive). Bodily entrainment conducive to the experience of empathy fundamentally involves haptic coupling and visual contact along with rhythmic movement together. This pilot study tested two research designs to demonstrate their usefulness in studying changes in closeness and empathy for partners who dance together in pairs, facing one another, while disentangling the effects of rhythm and coupling type. As a pilot study, it is intended to serve as a building block for further research on the origins of dance in mutual entrainment in the mother–infant dyad. An important limitation stems from the fact that the present study did not involve children or infants. However, a condition with mutual entrainment only is the closest one can get, as an adult, to a state that resembles the conditions of mother–infant interaction.

The analysis of IOS scores in the first experiment showed that there was no significant difference in how improvisation without external sound affected closeness by comparison with formalised dance where entrainment due to external regular rhythm is present in addition to mutual entrainment. The analysis also showed that a 7 min long dance/movement in pairs, whether improvising without external sound, or moving to a regular rhythm, significantly affected the feeling of closeness (F(1,22) = 17.405, *p* < 0.001), which increased similarly in both conditions. However, higher means for PT and F and lower means for EC and PD were found in Experiment 1 in the condition with regular rhythm compared to the condition with mutual entrainment only. While not statistically significant, this suggests that cognitive empathy may be stronger than affective empathy in dance with external rhythm. Conversely, higher EC and PD in the mutual entrainment condition suggests a predominance of affective empathy in this type of partnered interaction. These results are consistent with the theory that dance evolved from face-to-face interactions from both developmental and evolutionary perspectives ([18,19]). Should such results be strengthened in future studies, it would mean that, from an evolutionary perspective reflected in a developmental perspective, dance may have its origin in mutual entrainment without external sound, possibly in the first interactions between mother and infant, interactions which become socialised in childhood in rough-and-tumble play or forms of loosely formalised dance, and in adulthood in formalised dance. Thus, linear regression showed that higher EC pre-test values predict stronger increase in closeness with regular rhythm and full coupling (β = 0.766, *p* = 0.004), suggesting the possibility that ancestral humans may have noticed the phenomenon and used formalised dance in the hope of harnessing the power of empathic concern in order to ‘produce’ the prosocial benefits associated with closeness. Ancestral humans may have noticed, at folk psychology level, that mothers and infants with higher empathic concern achieve stronger closeness by moving together to a regular rhythm with full coupling. On the other hand, linear regression showed that closeness pre-test values predicted weaker increases in EC with regular rhythm (β = −0.579, *p* = 0.049), whereas the ANOVA showed stronger, if not statistically significant, increases in perspective taking and fantasy. Taking in both the ANOVA and regression results, one may speculate that cognitive empathy (PT and F) takes over the role of affective empathy (EC and PD) as a driver of closeness at some point, whether on the evolutionary or developmental scales, once movement to regular external rhythm with full coupling is mastered or learned, and practiced, requiring increased executive control.

Moreover, in the first experiment, the analysis of IRI subscale scores with time as a within-subjects factor found a significant effect of rhythm on empathic concern (F(1,22) = 4.375, *p* = 0.048). There was a statistically significant difference between the increase in empathic concern in the condition with improvisation (mutual entrainment only), and the decrease in EC when regular rhythm was present. In other words, having to find a rhythm together in movement improvisation had a stronger effect on empathic concern than following the formalised pattern of an external regular rhythm. This is consistent with the idea that affective empathy predominates in mother–infant mutual entrainment in the absence of external rhythm. It also once again suggests that, as the capacity for executive control increases, it may take over the role of affective empathy as the driver of the increase in closeness, since closeness increased similarly over time in both (rhythm) conditions.

In the second experiment, the factorial analysis using the difference between IOS post- and pre-trial scores showed a near-significant effect of rhythm type on closeness. There was a significant simple effect of the interaction of irregular rhythm with visual coupling and a near-significant simple effect of the interaction of irregular rhythm with full feedback, relative to the improvisation (mutual entrainment only) condition. Since the IOS mean values were lower for irregular rhythm, it appears that irregular rhythm had a significant adverse effect on closeness when partners could see each other face-to-face, whether or not they were holding hands, and, conversely, that improvisation (mutual entrainment only) more strongly fostered closeness with full or only visual coupling. In the broader perspective, this suggests that mutual entrainment is the pre-eminent process for engendering closeness in comparison with irregular rhythm.

It appears that, when taking into account the coupling type, holding hands with eyes closed during mutual entrainment did not help to foster closeness in comparison to visual coupling without holding hands, while visual coupling without touch contributed to the feeling of closeness nearly as much as visual coupling with touch (Figure 3). However, the analyses conducted with time as a within-subjects factor showed that closeness increased in all three coupling conditions, with no significant difference in this increase according to the coupling type (Figure 5), and a significant effect of the interaction between rhythm and time (F(2,45) = 3.842, *p* = 0.029) (Figure 4). Mean differences indicated that, in improvisation (mutual entrainment only), closeness increased more than twice as much as it did in the condition with regular rhythm, and that the increase with regular rhythm was, in turn, about twice as high as the increase with irregular rhythm (Table 3). Taken together, these results point to mutual entrainment (without external rhythm) as a powerful means of achieving closeness, but one that works best when visual coupling is also present. Even when EC pre-test values are higher, and haptic coupling is present, not seeing one partner seems to diminish the increase in closeness, as linear regression shows. This suggests that mutual entrainment by itself fosters closeness more than external rhythm, with the duration of the interaction and visual coupling as significant factors.

This is consistent with empirical observations of infant behaviour in mother–infant interaction. The fact that the same behaviour dynamics are present in adult dance suggests that mother–infant interaction and partnered dance at least belong in the same paradigm. In both cases, not seeing the other’s face may cause distress (see [20] for an EEG study), albeit distress that is consistent with affective empathy, as shown further below in the discussion of PD scores.

As regards IRI subscale measures, rhythm had a near-significant effect on empathic concern (F(2,43) = 3.140, *p* = 0.053), reflected in a near-significant difference between the condition with regular rhythm and the condition with improvisation, which occurred with visual coupling without hand holding (Figure 6). This echoes the findings of the first experiment, which evidenced an increase in empathic concern with improvisation relative to regular rhythm with full coupling (F(1,22) = 4.375, *p* = 0.048). Similarly, in the second experiment, empathic concern was stronger when partners improvised, picking up cues from each other visually, but this time without holding hands. Linear regressions suggest that higher pre-test IOS values predict a stronger increase in EC when regular rhythm is present and partners see each other without touching (β = 0.830, *p* = 0.041), whereas when haptic coupling is added, high pre-test IOS values predict a diminishing in the increase in EC. This could mean that haptic coupling is rather used for coordination, involving cognitive empathy (PT and F), when regular rhythm demands allocation of cognitive control resources for synchronisation. In the absence of reassuring touch, higher pre-test closeness seems to potentiate empathic concern. However, it should be noted that the data were collected from only six participants per condition in the second experiment, which calls for caution in interpreting the Experiment 2 regression results.

There was a significant effect of coupling type on fantasy: F(2,45) = 3.212, *p* = 0.050. Fantasy decreased when participants held hands, whether with their eyes closed or seeing each other, but it remained fairly constant when not holding hands while seeing each other (Figure 7). This suggests that visual coupling is involved in empathic fantasising, whereas fantasy decreases when haptic coupling is involved. This is likely an experience that occurs irrespective of whether the partners are adult or infants in the mother–infant dyad. While there are no studies of empathic fantasizing in face-to-face partnered movement, a case has been made in psychoanalysis and clinical psychology for fantasising as a main process of acquisition of subjectivity in infancy, which may continue in adulthood (e.g., [21]; for a possible interpretation of the relation between fantasising about one’s identity and taking another’s perspective on the self, see [22]). Intriguingly, the results of this study suggest that fantasising is stronger when adults do not touch, given that in psychoanalysis literature concerning infants and fantasising, a mother’s holding of the baby is a crucial element. Be that as it may, if fantasy is taken as a measure of cognitive empathy, it seems that it is stronger with visual coupling without touch, a situation that is predominant in adult interaction, than when touch, evoking mother–infant closeness, is present.

The coupling type also significantly affected personal distress: F(2,45) = 5.599, *p* = 0.007. There was a significant difference in means of personal distress scores between the condition with only haptic coupling and the conditions with full feedback and visual coupling alone. This variation in personal distress was stronger with irregular rhythm, and still quite strong with improvisation (Figure 8). When partners held hands with their eyes closed, personal distress was stronger than when they could see each other, whether or not they were holding hands, and strongest with irregular rhythm. This suggests that when irregular rhythm is present and partners hold hands with their eyes closed, they feel more personally distressed than when there is only mutual entrainment without external sound, and least distressed when regular rhythm is present. The repeated measures analysis again indicated an increase in personal distress when partners held hands with their eyes closed, and a decrease when they could see each other, whether or not they were holding hands. One might tentatively relate this result to the empirical observation that infants become distressed when they do not see their mother’s face, although adults may likewise become naturally distressed when they hold someone else’s hands with their eyes closed and have to coordinate their movements on an irregular rhythm.

The type of rhythm significantly affected perspective taking: F(2,40) = 3.293, *p* = 0.047. Specifically, there was a significant difference in perspective taking between the regular and irregular rhythm conditions involving touch, with much stronger use of perspective taking when irregular rhythm was present than when regular rhythm was present. There was also a stronger use of perspective taking during improvisation (mutual entrainment only) than when regular rhythm was present in the conditions involving touch (Figure 10). This suggests that, especially when irregular rhythm is present, partners seeing each other while holding hands, as well as partners holding hands with their eyes closed, are significantly engaged in perspective taking, seeking cues for moving together via haptic coupling (consistent with [11,12]). The same is true to a lesser extent during improvisation, that is, when partners find the rhythm of their movement using mutual cooperation, whereas regular rhythm involves less use of perspective taking, perhaps because of its predictability.

### 4.1. Conclusions

This pilot study sought to test two research designs to see whether they may be helpful in studies that could support the theory that dance originates in mutual entrainment in the mother–infant dyad from a developmental perspective and in mutual entrainment in prelinguistic/pre-cultural humans from an evolutionary perspective. The endeavour hinges on the hypothesis that, on the one hand, mutual entrainment predominantly fosters affective empathy (EC and PD) and is consistent with observed behaviour in the mother–infant dyad; on the other hand, partnered movement to a regular rhythm predominantly fosters cognitive empathy (PT and FS), and is consistent with observed behaviour in adult dancing partners. Since all four dimensions of empathy as conceived in the development of the IRI instrument are inextricable dimensions of global empathy, one cannot separate affective empathy from cognitive empathy. Rather, one might say that affective empathy is predominant in the mother–infant dyad and yields its privileged position to cognitive empathy in adult life, except in situations that demand greater intimacy. In this pilot study, improvisation without any external sound was regarded as demanding greater intimacy between adult partners moving together, and a set-up that more closely resembles the emotional dynamics taking place in the mother–infant dyad (or, indeed, among ancestral humans). It was thought that a case for locating the origins of dance in mutual entrainment in the mother–infant dyad might begin to be built if the results could suggest that affective empathy predominates in mutual entrainment only, and cognitive empathy predominates in entrainment to an external regular beat, even if all participants were adults. The tentative idea, glimpsed from lived experience, is that improvising adults behave more like children than adults dancing to a regular rhythm that requires more formally patterned movements.

The results indicating that empathic concern increases when partners improvise, seeking to pick up cues from their partner visually, support the perspectives outlined above. The finding that visual coupling is involved in empathic fantasy, whereas fantasy decreases when haptic coupling is involved, may likewise suggest that cognitive empathy is more strongly mobilised in adult interactions where less physical contact is normal than between mothers and their infants. The pilot study also found that one becomes distressed when holding hands with closed eyes for 7 min, and especially distressed if irregular rhythm is present, but also quite distressed when the partners improvise together. Personal distress is a dimension of affective empathy. If it is stronger with mutual entrainment, and, moreover, if mutual entrainment precedes entrainment to external rhythm developmentally and evolutionarily, then this result may again suggest that dance originates in mother–infant interactions, and adult dance perhaps evolved as a means of controlling such distress. Perspective taking may be used to alleviate distress: partners seeing each other while holding hands, but also partners holding hands with their eyes closed, significantly used perspective taking, especially with an irregular rhythm, perhaps also for coordination.

### 4.2. Limitations

The main limitations of this study are (1) that no infants were involved in the research, and (2) that the study was underpowered. Only about a third of the number of participants indicated by GPower analyses were recruited for the first experiment, while about half of the required participants for the second experiment were recruited. However, moderate to large effect sizes were obtained, along with a number of illuminating, statistically significant results. The interpretation of the regression results from Experiment 2 should be regarded with caution, as only six participants were recruited for each of the nine conditions of the second study.

## Figures and Tables

**Table 1:** Research Design 1 with 2 conditions: condition 1 resembles formal dances with full coupling (visual, auditory, and haptic) and external regular rhythm as well as mutual entrainment; condition 2 resembles improvisation movement with full coupling (visual, auditory, and haptic) and mutual entrainment only (i.e., without external sound).

	Visual	Auditory	Haptic
Condition 1 Regular external rhythm	Mutual + external entrainment	Mutual + external entrainment	Mutual + external entrainment
Condition 2 No external rhythm	Mutual entrainment only	Mutual entrainment only	Mutual entrainment only

**Table 2:** Research Design 2 with 9 conditions comprising all combinations of types of coupling, external rhythm, and mutual entrainment only. The coupling IV has 3 levels: full coupling (visual and haptic), haptic only, and visual only, and auditory coupling is subsumed under the rhythm IV. The rhythm IV comprises external rhythm (in turn comprising regular and irregular rhythm) and improvisation (condition with mutual entrainment only, without external rhythm). The arrows point to the respective IV levels.

IV Coupling→ IV Rhythm ↓	Visual, No Haptic	No Visual, Haptic	Visual and Haptic
**Regular rhythm (external + mutual entrainment)**	DVs: Empathy (IRI scores) and Closeness (IOS score)	DVs: Empathy (IRI scores) and Closeness (IOS score)	DVs: Empathy (IRI scores) and Closeness (IOS score)
**Irregular rhythm (external + mutual entrainment)**	DVs: Empathy (IRI scores) and Closeness (IOS score)	DVs: Empathy (IRI scores) and Closeness (IOS score)	DVs: Empathy (IRI scores) and Closeness (IOS score)
**No music (mutual entrainment only)**	DVs: Empathy (IRI scores) and Closeness (IOS score)	DVs: Empathy (IRI scores) and Closeness (IOS score)	DVs: Empathy (IRI scores) and Closeness (IOS score)

**Table 3:** Comparisons of estimated marginal means differences (M_dif_) between post- and pre-interaction means for all combinations of factor levels for IOS measures of closeness in Experiment 2. The arrows point to the respective IV levels.

IV Coupling → IV Rhythm ↓	Visual, No Haptic	No Visual, Haptic	Visual and Haptic
**Regular rhythm**	M_dif_ = 0.468	M_dif_ = 0.5	M_dif_ = 0.334
**Irregular rhythm**	M_dif_ = 0.167	M_dif_ = 0.333	M_dif_ = 0.167
**Mutual entrainment only (no rhythm)**	M_dif_ = 1.334	M_dif_ = 1.167	M_dif_ = 1.167

**Table 4:** Post hoc multiple comparison of means for IRI scores on the empathic concern scale showing a near-significant difference in means between improvisation (mutual entrainment only) and regular rhythm.

**Post Hoc Multiple Comparisons for the Effect of Rhythm Type**						
Dependent Variable: IRI Empathic Concern						
Tukey HSD						
(I) Rhythm	(J) Rhythm	Mean Difference (I–J)	Std. Error	Sig.	95% Confidence Interval	
					Lower Bound	Upper Bound
Regular rhythm + mutual entrainment	Irregular rhythm	−1.1176	0.61603	0.177	−2.6130	0.3777
	Mutual entrainment	−1.4706	0.61603	0.055	−2.9660	0.0248
Irregular Rhythm + mutual entrainment	Regular Rhythm	1.1176	0.61603	0.177	−0.3777	2.6130
	Mutual entrainment	−0.3529	0.62477	0.839	−1.8695	1.1636
Mutual entrainment only	Regular rhythm	1.4706	0.61603	0.055	−0.0248	2.9660
	Irregular rhythm	0.3529	0.62477	0.839	−1.1636	1.8695

Based on observed means. The error term is Mean Square(Error) = 3.318.

**Table 5:** Post hoc multiple comparison of means for IRI scores on the empathic concern scale showing that the strongest means difference occurred between the condition with regular rhythm and mutual entrainment and the condition with improvisation (mutual entrainment only), in combination with visual coupling only, in Experiment 2. ‘Full’ = full feedback (visual and haptic coupling).

Rhythm * Coupling					
Dependent Variable: IRI Empathic Concern					
Rhythm	Coupling	Mean	Std. Error	95% Confidence Interval	
				Lower Bound	Upper Bound
Regular rhythm + mutual entrainment	full	−1.000	0.744	−2.500	0.500
	visual	−1.167	0.744	−2.666	0.333
	haptic	−0.833	0.744	−2.333	0.666
Irregular rhythm + mutual entrainment	full	−1.000	0.815	−2.643	0.643
	visual	0.500	0.744	−1.000	2.000
	haptic	0.667	0.744	−0.833	2.166
Improvisation (mutual entrainment only)	full	0.333	0.744	−1.166	1.833
	visual	1.000	0.815	−0.643	2.643
	haptic	0.167	0.744	−1.333	1.666

**Table 6:** Post hoc means comparisons for types of coupling showing a significant difference between the condition with haptic coupling and the other two coupling conditions (full feedback and visual coupling) in Experiment 2.

**Multiple Comparisons**						
Dependent Variable: IRI Personal Distress						
Tukey HSD						
(I) Coupling	(J) Coupling	Mean Difference (I–J)	Std. Error	Sig.	95% Confidence Interval	
					Lower Bound	Upper Bound
full	visual	0.4444	0.94868	0.886	−1.8548	2.7437
	haptic	−2.5000 *	0.94868	0.030	−4.7992	−0.2008
visual	full	−0.4444	0.94868	0.886	−2.7437	1.8548
	haptic	−2.9444 *	0.94868	0.009	−5.2437	−0.6452
haptic	full	2.5000 *	0.94868	0.030	0.2008	4.7992
	visual	2.9444 *	0.94868	0.009	0.6452	5.2437

Based on observed means. The error term is Mean Square(Error) = 8.100. The symbol “*” indicates that the mean difference is significant at the 0.05 level.

**Table 7:** Multiple comparison of means for IRI scores on the personal distress scale showing the difference between the condition with regular rhythm and mutual entrainment and the conditions with irregular rhythm and mutual entrainment, and with improvisation (mutual entrainment only) in combination with haptic coupling in reference to visual coupling and full feedback (Experiment 2).

Rhythm * Coupling					
Dependent Variable: IRI Personal Distress					
Rhythm	Coupling	Mean	Std. Error	95% Confidence Interval	
				Lower Bound	Upper Bound
Regular + mutual entrainment	full	−0.333	1.162	−2.674	2.007
	visual	−0.500	1.162	−2.840	1.840
	haptic	−0.500	1.162	−2.840	1.840
Irregular + mutual entrainment	full	−2.667	1.162	−5.007	−0.326
	visual	−2.833	1.162	−5.174	−0.493
	haptic	3.000	1.162	0.660	5.340
Improvisation (mutual entrainment only)	full	−0.333	1.162	−2.674	2.007
	visual	−1.333	1.162	−3.674	1.007
	haptic	1.667	1.162	−0.674	4.007

**Table 8:** Simple main effects of each type of coupling within each level of the rhythm conditions, showing the significant simple effect of touch (haptic coupling only, with eyes closed) on the difference between personal distress with regular and irregular rhythm (*p* = 0.039). The improvisation condition is a condition without external sound and so with mutual entrainment only, whereas the regular and irregular rhythm conditions have external sound as well as mutual entrainment (Experiment 2).

**Pairwise Comparisons**							
Dependent Variable: IRI Personal Distress							
Coupling	(I) Rhythm	(J) Rhythm	Mean Difference (I–J)	Std. Error	Sig. ^b^	95% Confidence Interval for Difference ^b^	
						Lower Bound	Upper Bound
Full (visual + haptic)	regular	irregular	2.333	1.643	0.162	−0.976	5.643
		improvisation (no external rhythm)	−4.441	1.643	1.000	−3.310	3.310
	irregular	regular	−2.333	1.643	0.162	−5.643	0.976
		improvisation (no external rhythm)	−2.333	1.643	0.162	−5.643	0.976
	improvisation (no external rhythm)	regular	4.441	1.643	1.000	−3.310	3.310
		irregular	2.333	1.643	0.162	−0.976	5.643
Visual only	regular	irregular	2.333	1.643	0.162	−0.976	5.643
		improvisation (no external rhythm)	0.833	1.643	0.615	−2.476	4.143
	irregular	regular	−2.333	1.643	0.162	−5.643	0.976
		improvisation (no external rhythm)	−1.500	1.643	0.366	−4.810	1.810
	improvisation (no external rhythm)	regular	−0.833	1.643	0.615	−4.143	2.476
		irregular	1.500	1.643	0.366	−1.810	4.810
Haptic only	regular	irregular	−3.500 *	1.643	0.039	−6.810	−0.190
		improvisation (no external rhythm)	−2.167	1.643	0.194	−5.476	1.143
	irregular	regular	3.500 *	1.643	0.039	0.190	6.810
		improvisation (no external rhythm)	1.333	1.643	0.421	−1.976	4.643
	improvisation (no external rhythm)	regular	2.167	1.643	0.194	−1.143	5.476
		irregular	−1.333	1.643	0.421	−4.643	1.976

Based on estimated marginal means. ^b^ Adjustment for multiple comparisons: Least Significant Difference (equivalent to no adjustments). * indicates that the mean difference is significant at the 0.05 level.

**Table 9:** Post hoc multiple comparison of means for IRI scores on the perspective taking scale showing that the difference between irregular and regular rhythm significantly affected perspective taking in Experiment 2.

**Multiple Comparisons**						
Dependent Variable: IRI Perspective Taking scale						
Tukey HSD						
(I) Rhythm	(J) Rhythm	Mean Difference (I-J)	Std. Error	Sig.	95% Confidence Interval	
					Lower Bound	Upper Bound
Regular rhythm	Irregular rhythm	−1.7316 *	0.68868	0.042	−3.4078	−0.0554
	Improvisation (no external rhythm)	−1.3750	0.69903	0.134	−3.0764	0.3264
Irregular rhythm	Regular rhythm	1.7316 *	0.68868	0.042	0.0554	3.4078
	Improvisation (no external rhythm)	0.3566	0.68868	0.863	−1.3196	2.0328
Improvisation (no external rhythm)	Regular rhythm	1.3750	0.69903	0.134	−0.3264	3.0764
	Irregular Rhythm	−0.3566	0.68868	0.863	−2.0328	1.3196

Based on observed means. The error term is Mean Square(Error) = 3.909. The symbol “*” indicates that the mean difference is significant at the 0.05 level.

## Data Availability

Data is available as supplementary materials S3 and S4A–E.

## Supplementary Materials

The following supporting information can be downloaded at: https://www.mdpi.com/article/10.3390/bs13100859/s1, Soundtrack S1: Regular Rhythm 4-4; Soundtrack S2: Irregular Rhythm 7-8; Data S3: Design 1 UK Data; Data S4A: Design 2 Romania data IRI Empathic Concern; Data S4B: Design 2 Romania data IRI Fantasy; Data S4C: Design 2 Romania data IRI Personal Distress; Data S4D: Design 2 Romania data IRI Perspective-Taking; Data S4E: Design 2 Romania data IOS; S5: Table S1 Simple effects table showing a significant simple effect of visual coupling and a near significant simple effect of full feedback on closeness comparing the improvisation (mutual entrainment only) and irregular rhythm conditions, with significantly stronger closeness with improvisation (no external rhythm) (Experiment 2). Table S2 Estimated Marginal Means comparisons for each combination of levels of the rhythm and coupling factors at pre- and post- trial points for IOS scores in Experiment 2.
