# Supplementary material for: Pilot Studies on Empathy and Closeness in Mutual Entrainment/Improvisation vs. Formalised Dance with Different Types of Rhythm (Regular, Irregular, and No Rhythm) and Coupling (Visual, Haptic, Full Coupling): Building a Case for the Origin of Dance in Mutual Entrainment Empathic Interactions in the Mother–Infant Dyad
Source: Behav Sci (Basel). 2023 Oct 20;13(10):859. doi: 10.3390/bs13100859 (PMC10604627; doi:10.3390/bs13100859)
Supplement: Supplementary file 1 [file behavsci-13-00859-s001.zip › S5 Supplemental Materials (Tables).pdf]

Supplemental Materials (Tables)

Pairwise Comparisons (simple effects)
Dependent Variable: IOS

Coupling	(I) Rhythm	(J) Rhythm	Mean Difference (I-J)	Std. Error	Sig.[b]	95% Confidence Interval for Difference[b]	
						Lower Bound	Upper Bound
Full	Regular rhythm	Irregular rhythm	.167	.557	.766	-.955	1.289
		Improvisation (no external rhythm)	-.833	.557	.142	-1.955	.289
	Irregular rhythm	Regular rhythm	-.167	.557	.766	-1.289	.955
		Improvisation (no external rhythm)	-1.000	.557	.079	-2.122	.122
	Improvisation (no external rhythm)	Regular rhythm	.833	.557	.142	-.289	1.955
		Irregular rhythm	1.000	.557	.079	-.122	2.122
Visual only	Regular rhythm	Irregular rhythm	.302	.557	.591	-.820	1.424
		Improvisation (no external rhythm)	-.865	.557	.127	-1.987	.257
	Irregular rhythm	Regular rhythm	-.302	.557	.591	-1.424	.820
		Improvisation (no external rhythm)	-1.167*	.557	.042	-2.289	-.045
	Improvisation (no external rhythm)	Regular rhythm	.865	.557	.127	-.257	1.987
		Irregular rhythm	1.167*	.557	.042	.045	2.289
Haptic only	Regular rhythm	Irregular rhythm	.167	.557	.766	-.955	1.289
		Improvisation (no external rhythm)	.100	.584	.865	-1.077	1.277
	Irregular rhythm	Regular rhythm	-.167	.557	.766	-1.289	.955
		Improvisation (no external rhythm)	-.067	.584	.910	-1.244	1.110
	Improvisation (no external rhythm)	Regular rhythm	-.100	.584	.865	-1.277	1.077
		Irregular rhythm	.067	.584	.910	-1.110	1.244

Based on estimated marginal means
*. The mean difference is significant at the .05 level.
b. Adjustment for multiple comparisons: Least Significant Difference (equivalent to no adjustments).

Table S1: Simple effects table showing a significant simple effect of visual coupling and a near significant simple effect of full feedback on closeness comparing the improvisation (mutual entrainment only) and irregular rhythm conditions, with significantly stronger closeness with improvisation (no external rhythm) (Experiment 2).

Rhythm*Coupling*Time
Measure: Closeness

Rhythm	Coupling	time	Mean	Std. Error	95% Confidence Interval	
					Lower Bound	Upper Bound
Regular rhythm	Full	1	2.333	.593	1.139	3.528
		2	2.667	.556	1.548	3.786
	Visual only	1	2.500	.593	1.305	3.695
		2	2.968	.556	1.849	4.087
	Haptic only	1	2.833	.593	1.639	4.028
		2	3.333	.556	2.214	4.452
Irregular rhythm	Full	1	3.000	.593	1.805	4.195
		2	3.167	.556	2.048	4.286
	Visual only	1	1.667	.593	.472	2.861
		2	1.833	.556	.714	2.952
	Haptic only	1	2.000	.593	.805	3.195
		2	2.333	.556	1.214	3.452
Improvisation (mutual entrainment only)	Full	1	1.667	.593	.472	2.861
		2	2.833	.556	1.714	3.952
	Visual only	1	2.333	.593	1.139	3.528
		2	3.667	.556	2.548	4.786
	Haptic only	1	1.333	.593	.139	2.528
		2	2.500	.556	1.381	3.619

Table S2: Estimated Marginal Means comparisons for each combination of levels of the rhythm and coupling factors at pre- and post- trial points for IOS scores in Experiment 2.
